# Clinic of Professor Dunglison

**Published:** 1845-07

**Authors:** Samuel G. White

**Affiliations:** Georgia


### CLINICAL LECTURES AND REPORTS.

### PHILADELPHIA HOSPITAL.

*Saturday, February* 1, 1845.

### CLINIC OF PROFESSOR DUNGLISON.

Reported by Dr. Samuel G. White, of Georgia.

Professor Dunglison continued, this morning, the consideration of the class of nervous diseases, which engaged attention at the last lecture.

### PARAPLEGIA.

On this affection he offered only a few observations, as the inclemency of the weather rendered it hazardous to introduce the patients labouring under it into the amphitheatre; and he remarked that he made it a point that the patient should never incur any risk from being brought before the class.

This disease is seated in some portion of the medulla spinalis, which may generally be detected. It is characterized most usually by marked phenomena, which differ, however, in different cases. There is either impairment or abolition of the power of motion, or of sensation, or of both, in all the parts that receive their nerves from the medulla, below the seat of the disease. If there be paralysis alone, the anterior or motor nerves are affected only; if sensation be impaired or destroyed, the sensory or posterior nerves; and when sensation and motion together are injured, both roots are involved. The loss of the power of motion is detected earlier than that of sensation, the peculiar halting or vacillatory gait of the individual always attracting attention.

Paraplegia, then, may be caused by a lesion of any portion of the spinal medulla. It is very frequently the result of mechanical injuries, as of falls, blows, &c. The precise seat of the disease may generally be discovered, by the difference of sensation when pressure is made over the vertebræ, corresponding to, and below the seat of the lesion. The treatment, in fully formed paraplegia, essentially consists in the employment of stimulant applications; as the various counter-irritants, frictions, electricity, &c. Usually, however, more confidence is reposed in strychnia, given internally or applied endermically. If there be any apprehension as to the administration of this article in hemiplegia, the Professor thinks there should be none in paraplegia. Its excitant action in the former, when dependent on hemorrhage, may increase the effusion of blood, and occasion injurious effects on the encephalic neurine; but no such mischief need be feared in the affection under consideration. Strychnia is a tetanic, and, as such, exerts its influence on the nervous system, which is commonly evinced, more especially, by tetanic convulsions of the muscles of the affected limbs. It may be given in doses of the tenth of a grain, repeated twice or thrice daily, or the same quantity to one-sixth of a grain may be applied to a blistered surface over the affected portion of the spine. At times, the effect of this article is most salutary, but it is often productive of no benefit. Generally the case remains incurable when the medulla is organically diseased; and we can only rely for benefit on the recuperative powers of the system; which, however, are too often insufficient to repair the mischief.

The Professor then proceeded to make some remarks on

CHOREA.

This disease is classed amongst the neuroses, or those affections of the nervous system which are not characterised by any constant pathological lesion. As remarked at the last lecture, they afford good examples of the difficulty which exists, in some instances, in distinguishing, by nomenclature, symptoms from disease—in other words, the manifestations of the disease from the disease itself. Being ignorant of the pathological condition, we name the disease after a prominent manifestation.

Chorea, otherwise called *St. Vitus's dance*, is confined principally to children, although it does occur, at times, at an after period. It is usually attended with phenomena not easily to be mistaken. The principal of these is a constant, singular movement of some portion of the body. In rare instances, the motions involve the entire muscular system; but in the vast majority, they are confined to one side, and generally to the extremities; at times to the face, or to one upper extremity. Every variety of tic, or twitching of the face or muscles of the extremities, may with propriety be classed along with chorea. They are, indeed cases of *partial chorea;* are frequently the result of vicious habits, acquired at an early period of existence, and which have become inveterate, so as often to be real deformities.

The Professor now introduced a patient suffering with chorea, which, although not strongly marked, served to exhibit its characters. The patient, a young man, entered the hospital for scabies, for which he was treated. The history of the case being very imperfect, the nature of the disease could only be inferred from the existing symptoms, which were, constant motion of the right leg, twitching of one side of the face, and irregularity in locomotion. Soon after he entered the wards, he was attacked with convulsive movements of the left side without any affection of the mind, which were not well understood at the time, but seem evidently to have been augmented manifestations of chorea,—ordinary convulsions, excepting the hysteric and hysteroid, being attended with suspension of consciousness during their continuance. From the history obtained—imperfect as it is—it is probable that the chorea had existed before puberty, and has since continued.

The precise local pathology of chorea, as before intimated, is not known. There is a preternatural and peculiar excitability, and mobility of the nervous system, of an inappreciable character; but unquestionably dependent upon some organic modification, of the nature of which we are profoundly ignorant, as we are in the case of every other affection of the class neuroses.

It is stated that the left side is most frequently affected in chorea, but the numerical method has not thus far been rigorously applied, and it cannot be considered as determined.

It is a disease more often feigned than any other, especially in civil and military hospitals. Not unfrequently, the simulation is so perfect as to elude detection, unless careful attention be paid to the individual, when he considers himself unobserved.

The Professor here narrated an interesting case of this description that occurred in his own practice, and which he related to the clinical class of the last session. The patient—a young lady—from the incessant motion of one arm, was believed to be affected with chorea, and was subjected to every variety of treatment, but without effect, not the least suspicion being indulged of its real character. At the suggestion of an older physician, it was determined to watch the patient whilst secluded. Accordingly, the nurse was directed to notice her through the key hole of the room, when it was found, that, whilst alone, the arm remained perfectly quiet, but, on the least noise being made, it immediately commenced its wonted motions. The experiment was repeated several times with the same result, and the inference was that the affection was feigned. On farther inquiry, suggested by these investigations, the girl confessed, that being dissatisfied with her situation at a boarding shool, she had adopted this method of being retained at home.

It has been affirmed, that the motions, in the feigned form of the disease, are discontinued during sleep, and that by this test its character may be ascertained; but in many cases of real chorea there are no movements whatever in sleep, and hence it cannot be considered distinctive. The fraud can, indeed, only be discovered by pursuing a plan similar to the one alluded to, of watching the individual closely.

The prognosis is somewhat modified by the age of the patient. If under puberty, it is always more manageable, and is, in many cases, spontaneously removed under the evolutions that take place in the system at that period. But if it continues beyond this, it is very apt to persist, in spite of any thing that can be done.

It is not, generally speaking, a serious disease, but at times the functions of the encephalon become disturbed, and want of cerebral power or idiocy results. The treatment has varied according to the peculiar views of practitioners in regard to the nature of the affection. They, who consider it to be phlogistic, have recourse to the antiphlogistic regimen, and to general or topical blood-letting, cathartics, low diet, &c. Dr. Hamilton, sen., of Edinburgh, believing it to be dependent on torpor of the gastro intestinal surface, placed much confidence on the continued use of purgatives; and we can readily comprehend, that offending matters existing in the intestines, might react injuriously in the nervous system, and be concerned in the production of the phenomena. Under such circumstances, their removal could not fail to be beneficial, and, doubtless, in this manner cathartics may often be productive of much good. The Professor does not consider it necessary to employ depletory means in the majority of cases, as he believes the disease to be anything but inflammatory. They may, however, be sometimes required. He thinks, that most benefit results from a combination of cathartics with tonics. There is great irritability and mobility of the nervous system in every case, which must be removed in order to effect a cure. Of the tonics, the preparations of iron, and especially the subcarbonate, are to be preferred.

In the case under consideration, a purgative, consisting of jalap and calomel, shall be given every third day; and in the interval the subcarbonate of iron—in large doses—not less than twenty to twenty-five grains, three times a day. These articles require to be continued for several weeks, before any marked effect is usually produced, and the same may be said of any mode of treatment for chorea. The result in this case will be reported to the class on some future occasion. Where practicable, these agents may be aided by the use of the shower-bath in summer, frictions with the flesh-brush, travelling air and exercise, change of residence, &c., every thing, indeed, which is adapted to make a new or revellent impression on the nervous system, may be had recourse to, and decided benefit will often result; but, at times, the disease is exceedingly obstinate, and—as before remarked—resists every mode of treatment.

In connection with this subject, the Professor referred to an interesting case, of what was considered to be

### SALAAM CONVULSION.

This is a peculiar disease, characterised by a constant bobbing of the head forwards, which increases in degree until the whole body is flexed on the legs; and from its resemblance to the mode of salutation in the East, Sir Charles Clarke gave the name "*Salaam*" to it. The recorded cases of it are few, and they have been unfortunate in their termination, having either ended in idiocy or death.

The case, narrated by the lecturer, was contained in a letter from a professional gentleman. It described the case of his son. The lecturer did not, however, consider that it was truly a case of Salaam Convulsion. He feared that the phenomena—convulsions without loss of consciousness, and transient hemiplegia—indicated some alarming lesion of the cerebral neurine, the exact nature of which could not be ascertained. Such eccentric convulsions, he need hardly say, should be looked upon with great solicitude. An occasional convulsion, caused by eccentric impressions, may be of comparatively little moment; but one which originates from a morbid condition of the neurine, is apt to recur, to increase rather than diminish, and to induce unhappy consequences. He hoped that such might not be the result in the case in question, but he much feared it.

All the cases of salaam convulsion, hitherto described, have terminated unfavourably, the individual becoming idiotic or paralytic, and rarely surviving the 17th year. The disturbance may, in the first instance, be only functional; but, sooner or later, organic alterations in the encephalic neurine supervene.

The constant bobbing of the head forward recalls to mind the view of Magendie, that a forward impulse is seated in the cerebellum; and a backward in the corpora striata. According to this view, some lesion ought to exist in the seat of the backward impulse! But thus far we know nothing of the precise encephalic lesions in this singular affection.

### DISCOLORATION FROM NITRATE OF SILVER.

The attention of the class was next directed to a case of Epilepsy, not so much to illustrate that affection, as to exhibit the peculiar discoloration of the surface generally ascribed to the prolonged use of the nitrate of silver. The patient before the class, a middle aged man, has long suffered from epileptic convulsions. Whilst a child, he received a kick from a horse, and, five years afterwards, the fits made their appearance. It would not seem improbable that they are dependent on this cause, although the effect was not produced immediately. Perhaps this injury gave rise to the formation of an exostosis on the inner table of the skull, which we know is always gradual in its formation, and disturbs the functions of the brain, by irritation and pressure on the surface of that viscus. Not unfrequently, epilepsy is produced by a depressed fracture or splintered bone, which acts in a similar manner. The Professor remarked, that on another occasion he should consider this disease fully; and therefore at present would not say more upon it.

The patient before the class had been subjected to a great variety of treatment for the epilepsy without effect, and amongst other articles, it is probable, that the nitrate of silver had been prescribed. The peculiar colour of the skin would certainly lead to the inference, that this salt had been taken. It is affirmed, however, by some, that the slate colour of the surface, in such cases, is the effect of the disease, and not of the remedy. This view is held especially by those who adhere to the exploded opinion, that active substances cannot be received into the circulation. But if the discolouration be the effect of the disease, why is it that every patient does not present it. Why is it that we meet with such a small number of cases? In every instance, the lecturer remarked, in which the colour had been observed—so far as he knew—the nitrate of silver had been administered, and he is not aware of any authenticated casé in which it had occurred where the nitrate had not been given.

It is an interesting question to determine the *modus operandi* of the salt of silver in producing this discoloration. Is it converted, in the stomach, into a chloride? There is reason to believe, that it always must be, as that organ generally contains more or less chlorohydric acid, or chloride of sodium. This necessary decomposition is not, however, assented to by all, some believing that there is no change produced on the article in the stomach; but from the facility with which it is changed into the chloride out of the body, it is difficult to conceive how so small a quantity as the usual dose can possibly escape decomposition in the alimentary tube. The chloride, by exposure to light, becomes of a dark hue, or slate colour, which is so far confirmatory of its being the colouring agent in these cases.

The Professor infers, therefore, that the chloride is taken up from the gastro-interic mucous surface, and deposited in the corpus papillare, where it undergoes a change under the influence of light, so as to produce the colour described. The use of iodine along with the nitrate, is said to prevent this discoloration; or the iodide of silver may be administered in its place in epilepsy. The discoloration is not of frequent occurrence, but when it takes place it is not easily removed, and at times continues through life. Of late, Dr. Patterson, of Scotland, has recommended the preparations of iodine to remove the stain, and they *may* be beneficial. Sufficient trials have not been made with them, however, to establish their efficacy.

The iodide of potassium will be ordered in this case, and its results be reported to the class hereafter. It need scarcely be said that the remedy, to be of any service, must be continued for a considerable length of time.

### PATHOLOGICAL SPECIMENS.

Professor Dunglison next exhibited to the class an interesting and instructive case of

### OPEN FORAMEN OVALE, AND PULMONARY CONSUMPTION, IN A COLORED FEMALE NINETY-EIGHT YEARS OF AGE.

It will be remembered, that a communication between the auricles, through the septum auriculorum, exists throughout the term of utero-gestation, and for a time after birth. Such communication is all important to the proper performance of the fœtal circulation; but so soon as respiration is established, the blood is drawn into the lungs, and, the necessity for the foramen ovale ceasing, it closes.

The precise period at which this closure takes place is various. Sometimes it remains patulous for months, and, in a few instances, of which the case under consideration was a remarkable example, it continues until very advanced age.

Where there is no impediment to the pulmonic circulation, this open state of the foramen ovale is of no importance, and occasions no inconvenience, but when any impediment exists, CYANOSIS or MORBUS CÆRULEUS may be produced.

The Professor considers this affection—cyanosis—to be dependent not on a non-closure of the foramen, but generally rather on the imperfect development of the pulmonary artery, which prevents the free exit of the blood from the heart to the lungs. Any thing, indeed, that obstructs the pulmonic circulation may be its cause. Cyanosis, consequently, does not necessarily occur when the foramen is patulous. Of this, numerous cases are on record; and in the present case there was no evidence whatever, during life, of cardiac malformation, although the opening is sufficiently large to admit the handle of a medium sized scalpel.

It is not uncommon to meet with an open foramen in young individuals, but it is more rarely seen in adults. When it does exist, there are usually other malformations, which occasion the diseases of morbus cæruleus, so that life is seldom prolonged beyond the twelfth year. This is probably owing to the great susceptibility to morbid action, occasioned by the impeded circulation, and the consequent disturbance of the function of nutrition. Before the twelfth year the functions may be carried on, notwithstanding such impediments, but the evolutions that occur about the age of puberty may require blood more perfectly constituted, and a more perfect circulation, and hence the fatal disorders that generally supervene at this age in the cyanosed.

When cyanosis is dependent on an impediment to the egress of the blood from the heart, owing to an imperfectly developed pulmonary artery, &c., Dr. Craigie suggests, that the open condition of the foramen ovale may be even advantageous; inasmuch as the ventricles might otherwise be over-distended, and the blood not find a sufficient vent through the septum auriculorum. Life is not, therefore, destroyed by the admixture of arterial and venous blood, but is positively prolonged by it.

[Here the Professor exhibited the heart to the class, and pointed out the seat and size of the opening.]

The aorta in this case, as it generally is in old individuals, is studded with patches of osseous matter. These cannot be properly considered morbid, for ossification of the arteries becomes, as it were, a natural condition of those vessels in the aged. Very rarely is it absent, under such circumstances. The left heart presents a healthy appearance, with the exception of ossific points on the semilunar valves. This probably occasioned a roughness of both sounds of the heart, but especially of the second, which might have been detected, if auscultation had been practised. In the right heart, the tricuspid valves are markedly insufficient, and must have permitted the ready regurgitation of the blood from the ventricle to the auricle. The chordæ tendineæ are also very small.

It may here be remarked, that the right side of the heart is rarely diseased, whereas the left is very frequently so. This difference is, doubtless, connected with the difference in the texture of the lining membrane of the two sides,—that of the left being continuous with that of the aorta, whilst that of the right is a continuation of the venous membrane of the venæ cavæ. The semilunar valves of the pulmonary artery, and that vessel itself, are perfectly healthy.

*Lungs.*—The lungs were found adherent, in several places, to the chest, by fibrous bands, the result of ancient pleurisy. The right lung was the seat of extensive tubercular deposits, and near its apex was an excavation, large enough to contain several ounces of fluid, and another of smaller size, situate lower down, which was filled with softened tuberculous matter. The left lung was much congested, but contained no tubercles, or a very few. The bronchial glands were enlarged and softened.

The cavities of the heart and great vessels were filled with coagula of dark blood, and the substance of the organ was much engorged. From the sudden death of the patient, and the *post mortem* appearances, the lecturer concluded, that the cause of death was probably an arrestation of the heart's action, from its congested condition, consequent on impediment to the pulmonic circulation.

[The lecturer here presented another specimen of OPEN FORAMEN OVALE, occurring in an adult about thirty years of age, who died of cirrhosis of the liver. The opening, in this instance, was not so marked as in that just described, and was more valvular in its arrangement.]

The lecture was concluded with a few remarks on some pathological specimens, obtained from an individual under treatment for

ULCERATION OF THE RECTUM.

The case was reported on a former occasion.

Death occurred ultimately from phthisis pulmonalis. It is thought by some that ulceration of the rectum and fistula in ano, occurring in phthisical patients, have an intimate connection with the condition of the lungs. The Professor thinks, that this relation of cause and effect does exist to some degree, but not to such an extent as has been believed by many. Ulcerations of the intestines are frequently the consequence of pulmonary disease; but these are generally seated in the small intestine; but that the same connection with fistula obtains, he thinks doubtful.

The lungs in this case, as the class will see, are greatly hypertrophied, and completely solidified at the upper portion. The right lung, near its summit, is loaded with tubercles, and near the apex, posteriorly, a cavity of some size exists, from which the matter of expectoration was discharged. The opposite lung contains only a few tubercles of small size.

Ulcerations of different sizes are perceptible in the colon and rectum. In the large intestine they are numerous, and appear to have been of long standing. Although much inconvenience was experienced during life in the rectum, the ulcerations there seated are not so large or numerous as higher up. This condition of the parts affords a satisfactory explanation of the long continued diarrhœa under which the individual laboured.

